# 21 Disease activity and body mass index in juvenile idiopathic arthritis: is the truth revealed?

**DOI:** 10.1093/rheumatology/keac496.017

**Published:** 2022-10-07

**Authors:** H Ferjani, L Kharrat, M Moalla, W Triki, D Ben Nessib, K Maatallah, D Kaffel, W Hamdi

**Affiliations:** 1 Rheumatology Department, Kassab Orthopedics Institute, Ksar Saïd, Tunisia; 2 Faculty of Medicine, University Tunis el Manar, Tunisia

## Abstract

**Background:**

Active disease in chronic rheumatic diseases in adults seems to be associated with overweight and obesity [1]. Juvenile Idiopathic Arthritis (JIA) is the most frequent rheumatic disease in children. The relationship between body mass index (BMI) and disease activity in JIA patients is less studied.

**Objective:**

To determine the link between BMI and disease activity in JIA patients.

**Methods:**

We conducted a cross-sectional study including 35 JIA patients meeting the International League of Associations for Rheumatology (ILAR) 2001 criteria.

For each patient, we collected the following data: age, age at the onset of JIA, disease duration, patient global assessment (PGA), visual analogic scale (VAS), tender joint count (TJC), swollen joint count (SJC), disease activity using the Juvenile Arthritis Disease Activity score (JADAS), and therapeutic management.

The weight and height were measured for each patient. The BMI was calculated and interpreted according to the World Health Organization classification.

We also measured C-reactive protein (CRP) and Erythrocyte sedimentation rate (ERS) level.

**Results:**

We included 15 boys and 20 girls. The mean age was 12.2 ± 3.61 years. The mean age at the onset of the disease was 8.65 ± 3.83 years. The mean disease duration was 4.1 ± 3.29 years.

The mean PGA and the mean VAS were 3.4 ± 3.02 and 3.37 ± 2.92, respectively.

The mean TJC and the mean SJC were 1.48 ± 1.69 and 0.61 ± 0.77, respectively.

The mean CRP and ESR were 7.51 ± 11.85 mg/l and 18.88 ± 15.53 mm, respectively.

The mean JADAS was 7.58 ± 6.3. Twenty-four patients were under non-steroidal anti-inflammatory drugs (69%), 10 patients were under methotrexate (34%), and 5 patients were under TNFα inhibitor (14%).

The mean BMI was 19.94 ± 5.46 kg/m². Fourteen patients were underweight (40%), 15 patients had a BMI in the normal range (43%), 4 patients were overweight (11%), and 2 patients were obese (6%).

Underweight patients had higher PGA, higher CRP level, higher tender joint count (TJC), and higher JADAS compared with overweight and obese patients, but without significant difference (PGA: 2.97 ± 2.6 *vs* 2 ± 1.89, *p* = 0.4; CRP: 7.54 ± 15.09 *vs* 4.25 ± 4.19 mg/l, *p* = 0.6; TJC: 1.7 ± 1.56 *vs* 1 ± 0.8, *p* = 0.3, and JADAS: 6.3 ± 6.18 *vs* 2.98 ± 2.24, *p* = 0.1).

No correlations were found between BMI and the following parameters: TJC, SJC, EGP, CRP, ESR, and JADAS.

**Conclusion:**

Data about associations between underweight, obesity, and disease activity in JIA are conflicting. Some authors suggested that active disease is associated with adiposity [2]. Others showed that underweight patients had higher disease activity [3]. Although there was no significant difference, our study is consistent with Neto *et al.* study. Indeed, active disease seems to affect the child’s appetite and weight gain leading to cachexia [3]. Other studies are needed to confirm these results.

**References**

1. Bindesbøll C, Garrido-Cumbrera M, Bakland G, Dagfinrud H. Obesity Increases Disease Activity of Norwegian Patients with Axial Spondyloarthritis: Results from the European Map of Axial Spondyloarthritis Survey. Curr Rheumatol Rep. 2020; 22(8):43.

2. Diaz-Cordovés Rego G, Núñez-Cuadros E, Mena-Vázquez N, Aguado Henche S, Galindo-Zavala R, Manrique-Arija S, *et al.* Adiposity Is Related to Inflammatory Disease Activity in Juvenile Idiopathic Arthritis. J Clin Med. 2021; 10(17):3949.

3. Neto A, Mourão AF, Oliveira-Ramos F, Campanilho-Marques R, Estanqueiro P, Salgado M, *et al.* Association of body mass index with Juvenile Idiopathic Arthritis disease activity: a Portuguese and Brazilian collaborative analysis. Acta Reumatol Port. 2021; 46(1):7–14.

